# The Surgeon's Hand: The Most Sensitive Instrument in Detection of Small Bowel Neuroendocrine Tumors

**DOI:** 10.1111/ans.70487

**Published:** 2026-01-20

**Authors:** Jessica Falon, Krishna Kotecha, Nick Pavlakis, Anthony J. Gill, Anubhav Mittal, Jaswinder S. Samra

**Affiliations:** ^1^ Department of Upper Gastrointestinal Surgery Royal North Shore Hospital St Leonards New South Wales Australia; ^2^ Northern Clinical School, Faculty of Medicine and Health University of Sydney Sydney Australia; ^3^ Department of Medical Oncology Royal North Shore Hospital St Leonards New South Wales Australia; ^4^ NSW Health Pathology, Department of Anatomical Pathology Royal North Shore Hospital St Leonards New South Wales Australia; ^5^ Cancer Diagnosis and Pathology Group Kolling Institute of Medical Research, Royal North Shore Hospital St Leonards New South Wales Australia; ^6^ School of Medicine University of Notre Dame Sydney Australia

**Keywords:** hand‐assisted laparoscopy, minimally invasive surgical procedures, neuroendocrine tumors, small intestine, upper gastrointestinal tract

A 72‐year‐old man presented with post‐prandial abdominal pain and constipation. Computed tomography (CT) demonstrated a spiculated desmoplastic mesenteric mass consistent with a metastatic small bowel neuroendocrine tumor (NET), without a clear primary lesion (Figure 1A). ^68Ga‐DOTATATE positron emission tomography (PET) revealed an avid mesenteric deposit and several faint foci within small bowel loops, interpreted as possible nodal disease (Figure 1B), and FDG‐PET redemonstrated only the mesenteric lesion (Figure 1C). Following multidisciplinary discussion, the decision was made for surgery. At laparoscopy, the mesenteric lesion was identified, but the adjacent small bowel appeared macroscopically normal. A mini‐laparotomy was performed at the umbilicus for extracorporeal assessment, resection, and anastomosis of the small bowel, upon which at least eight multifocal tumors were palpable (Figure 2). Histopathology confirmed 13 well‐differentiated neuroendocrine tumors (3–12 mm) with nodal metastases, perineural invasion, and lymphovascular invasion (pT2 pN2).

**FIGURE 1:**
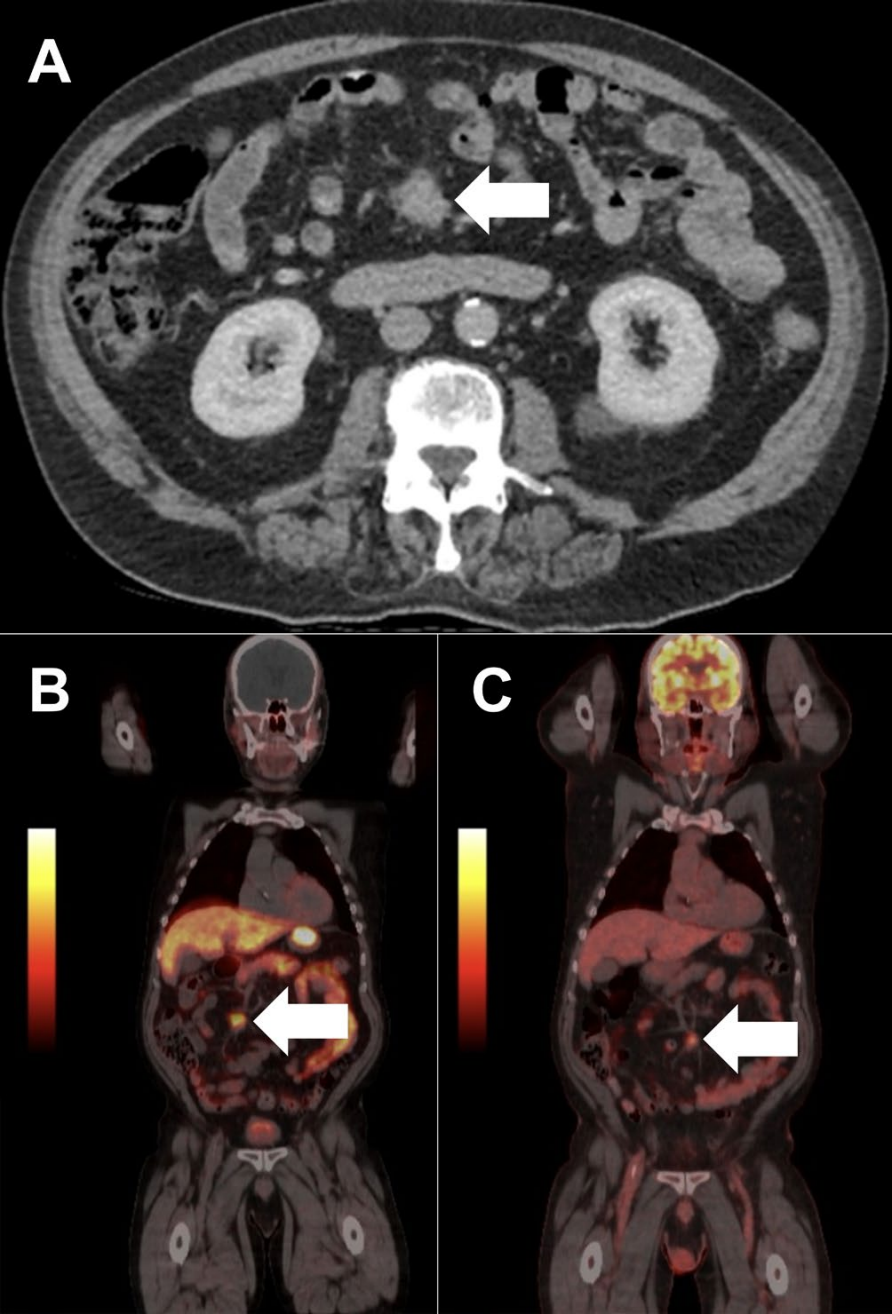
(A) Computed tomography (CT), (B) ^68Ga‐DOTATATE positron emission tomography (PET), and (C) FDG‐PET demonstrating an avid mesenteric deposit (white arrows), in the absence of a clear primary lesion.

**FIGURE 2:**
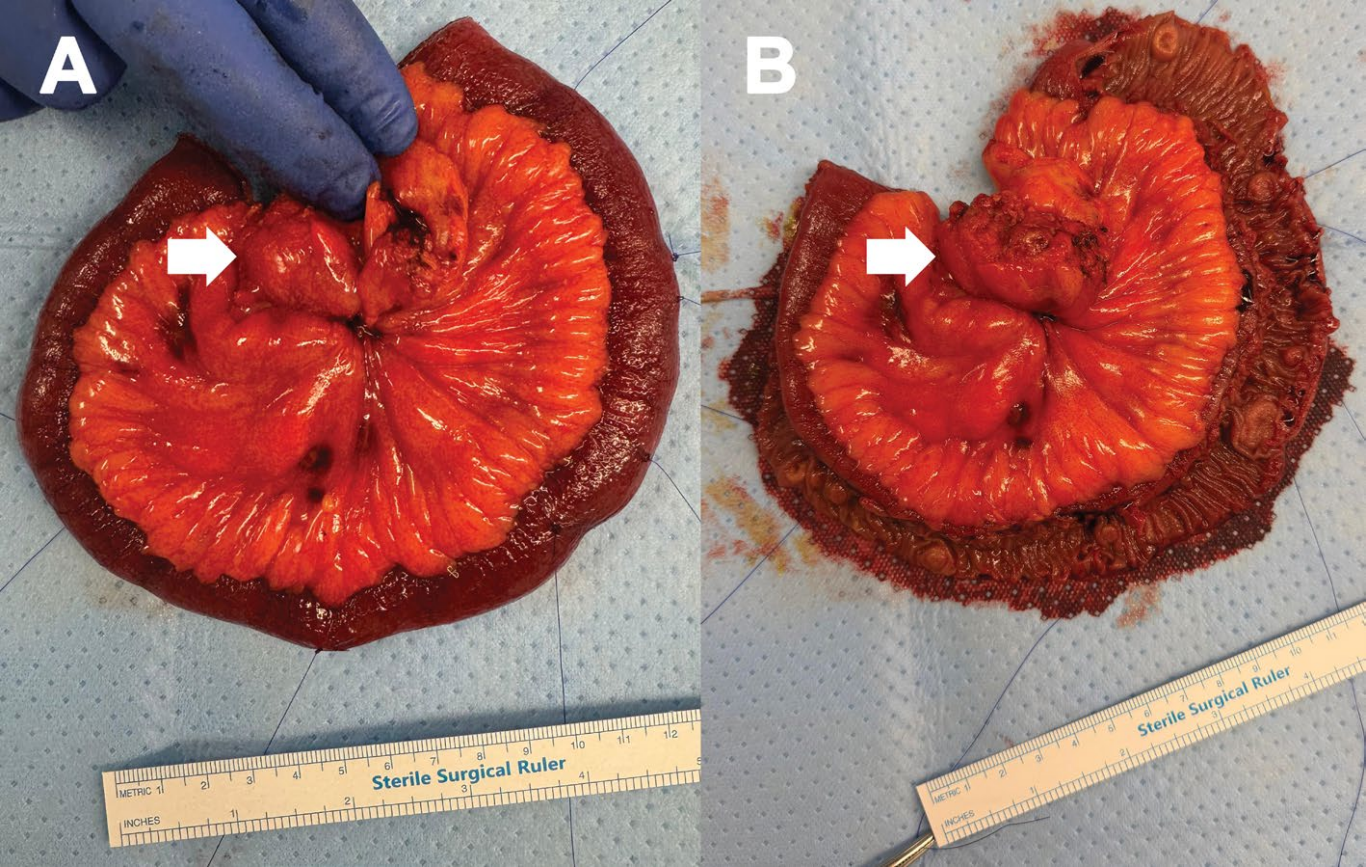
Intraoperative image demonstrating a cicatrizing mesenteric lesion (white arrows), with appearance of (A) a macroscopically normal serosa despite (B) multiple mucosal small bowel lesions.

Multifocality is a well‐defined feature of small bowel NETs, reported in 30%–50% of cases [1, 2]. Pre‐operative imaging such as CT and ^68Ga‐DOTATATE PET are only able to detect 12% and 47.5% of small bowel NETs, respectively [3]. In this case, lesions missed on imaging and laparoscopy were identified only by manual palpation. Consensus Guidelines of the North American Neuroendocrine Tumor Society currently advocate for exploratory laparotomy as the surgical intervention of choice for small bowel NETs, with careful manual palpation of the entire small bowel to identify small or multifocal lesions [4]. A laparoscopic approach, while conferring improved postoperative recovery [5, 6, 7], carries a risk of missing smaller synchronous lesions that are not easily palpable with laparoscopic instruments due to the lack of tactile feedback. Studies suggesting a benefit to overall survival with laparoscopy are likely confounded by selection of patients with early‐stage and less multifocal disease [7, 8].

By contrast, a laparoscopy‐assisted approach allows laparoscopic exploration of the abdomen with detailed inspection for metastatic disease, while facilitating manual palpation of the small bowel via a smaller incision than that required for a laparotomy. While outcomes between open and laparoscopy‐assisted resection have not been well‐studied, one retrospective cohort, single‐center study found that assisted laparoscopy demonstrated similar operative time, length of stay, and rates of radical resection in patients with stage II–III disease; however, it reduced postoperative analgesia requirements compared to open procedures [9]. Given that multifocal primary disease is frequently centered around regional lymph node metastases [10], a laparoscopy‐assisted approach may be preferred for patients with evidence of regional metastases on preoperative imaging.

Ultimately, the surgeon’s hand remains the most sensitive instrument in the detection of small bowel NETs. While minimally invasive surgery offers clear recovery advantages, tactile assessment of the small bowel remains indispensable for complete oncologic clearance. A laparoscopy‐assisted approach, allowing exteriorisation and palpation of the bowel, may preserve these benefits while ensuring detection of multifocal disease. Further studies directly comparing an open to a laparoscopy‐assisted approach, particularly with respect to long‐term survival and recurrence rates, are needed to inform guidelines for optimal surgical management of small bowel NETs.

## Author Contributions

**Jessica Falon:** writing – original draft, data curation, visualization; **Krishna Kotecha:** data curation, conceptualization, investigation, writing – review and editing, supervision; **Nick Pavlakis:** conceptualization, investigation, methodology, writing – review and editing; **Anthony J. Gill:** conceptualization, investigation, methodology, writing – review and editing; **Anubhav Mittal:** conceptualization, investigation, methodology, writing – review and editing; **Jaswinder S. Samra:** conceptualization, investigation, methodology, writing – review and editing, supervision.

## Disclosure

All authors are in agreement with the content of the manuscript. The manuscript has not been published previously and is not under consideration elsewhere.

## Ethics Statement

The authors have nothing to report.

## Consent

Written informed consent was obtained from the patient for publication of this case report and any accompanying images. All reasonable efforts have been made to protect patient anonymity.

## Conflicts of Interest

A.J.G. has received honoraria and travel support from AstraZeneca, Daiichi Sankyo, and Servier for work on topics outside the scope of this work. K.K. is the recipient of the Royal Australasian College of Surgeons John Loewenthal Grant, the Medtronic Pancreatic Surgery Fellowship, and the Australian Government Research Training Program offset.

## Data Availability

Data sharing not applicable to this article as no datasets were generated or analyzed during the current study.
